# Disseminated *Saksenaea* infection in an immunocompromised host associated with a good clinical outcome: a case report and review of the literature

**DOI:** 10.1186/s12879-020-05459-9

**Published:** 2020-10-14

**Authors:** N. Davidson, K. Campbell, F. Foroughi, V. Tayal, S. Lynar, L. C. Crawford, S. E. Kidd, R. Baird, J. Davies, E. M. Meumann

**Affiliations:** 1 Division of Medicine, Royal Darwin Hospital, Darwin, Australia; 2 Sullivan and Nicolaides Pathology, Brisbane, Australia; 3 Department of Pathology, Royal Darwin Hospital, Darwin, Australia; 4 Global and Tropical Health Division, Charles Darwin University, Menzies School of Health Research, Darwin, Australia; 5 National Mycology Reference Centre, Microbiology and Infectious Diseases, SA Pathology, Frome Road, Adelaide, South Australia Australia

**Keywords:** Mucormycosis, Disseminated fungal infection, Amphotericin B, Posaconazole

## Abstract

**Background:**

*Saksenaea* species (spp.) are uncommon causes of mucormycosis but are emerging pathogens mostly associated with trauma and soil contamination often in immunocompetent hosts. Due to lack of sporulation in the laboratory, diagnosis and susceptibility testing is difficult so optimal treatment regimens are unknown.

**Case presentation:**

A 67 year-old man from the Northern Territory in Australia, with a history of eosinophilic granulomatosis with polyangiitis, developed disseminated *Saksenaea* infection after initially presenting with symptoms consistent with bacterial pyelonephritis. Despite a delay in diagnosis; with aggressive surgical management and dual therapy with amphotericin B and posaconazole, he survived.

**Conclusions:**

We describe an unusual case of disseminated infection with a favourable outcome to date.

## Introduction

*Saksenaea* spp. are of the order *Mucorales* and are infrequent causes of mucormycosis worldwide [1]. *Saksenaea* spp. were first described in 1953 (as *Saksenaea vasiformis*) from soil in India and since then have been increasingly reported in human disease causing a diverse spectrum of clinical illness mostly in tropical and subtropical regions [2]. With the advent of molecular typing, multiple species have been described within the genus including *S. vasiformis*, *S. erythrospora*, *S. oblongispora, S. loutrophoriformis, S. trapezispora and S. dorisiae* [3–5]*.* In contrast to other causes of mucormycosis, *Saksenaea* spp*.* infections often cause soft tissue or bone and joint infections in immunocompetent hosts following traumatic inoculation. Rhinosinusitis and disseminated disease are much less common but have been associated with poor outcomes with survival reported in only two previous cases of disseminated infection [1]. We describe a case of disseminated infection by a *Saksenaea* species with a good clinical outcome and highlight rheumatological conditions as under-recognised risk factors for mucormycosis.

## Case report

A 67 year-old male living in tropical northern Australia presented with left sided abdominal pain, fever and pyuria. Computed tomography (CT) demonstrated abnormal enhancement of the lower pole of his left kidney, thickened pelvic urothelium and perinephric fat stranding. A provisional diagnosis of pyelonephritis was made, and he was empirically treated with intravenous ampicillin and gentamicin. His past medical history was significant for recurrent sinusitis with eosinophilia and positive perinuclear antineutrophil cytoplasmic antibodies, and he was on treatment for eosinophilic granulomatosis with polyangiitis (EGPA) with mycophenolate mofetil 750 mg twice daily and prednisone 5 mg daily.

**Fig. 1:**
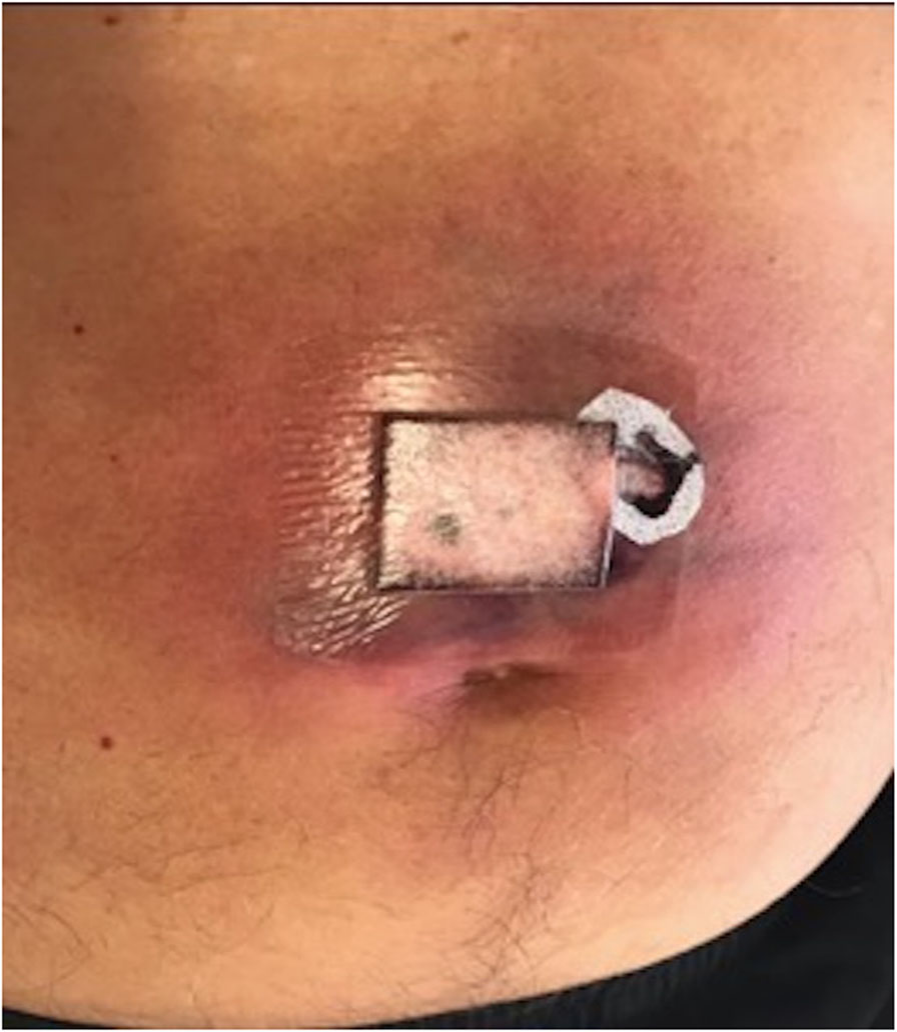
Abdominal wall lesion

**Fig. 2:**
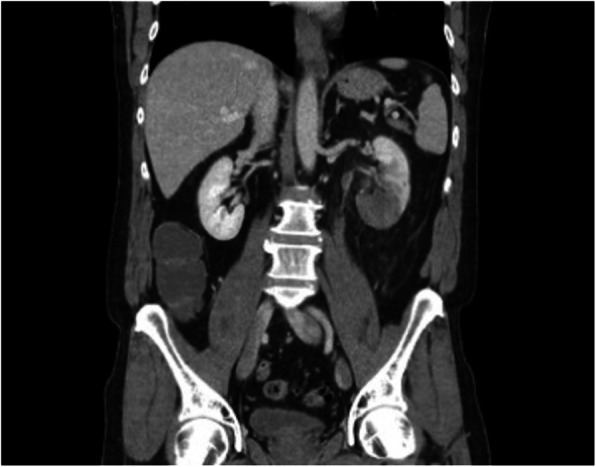
CT scan demonstrating absence of perfusion in the lower pole of the left kidney

**Fig. 3:**
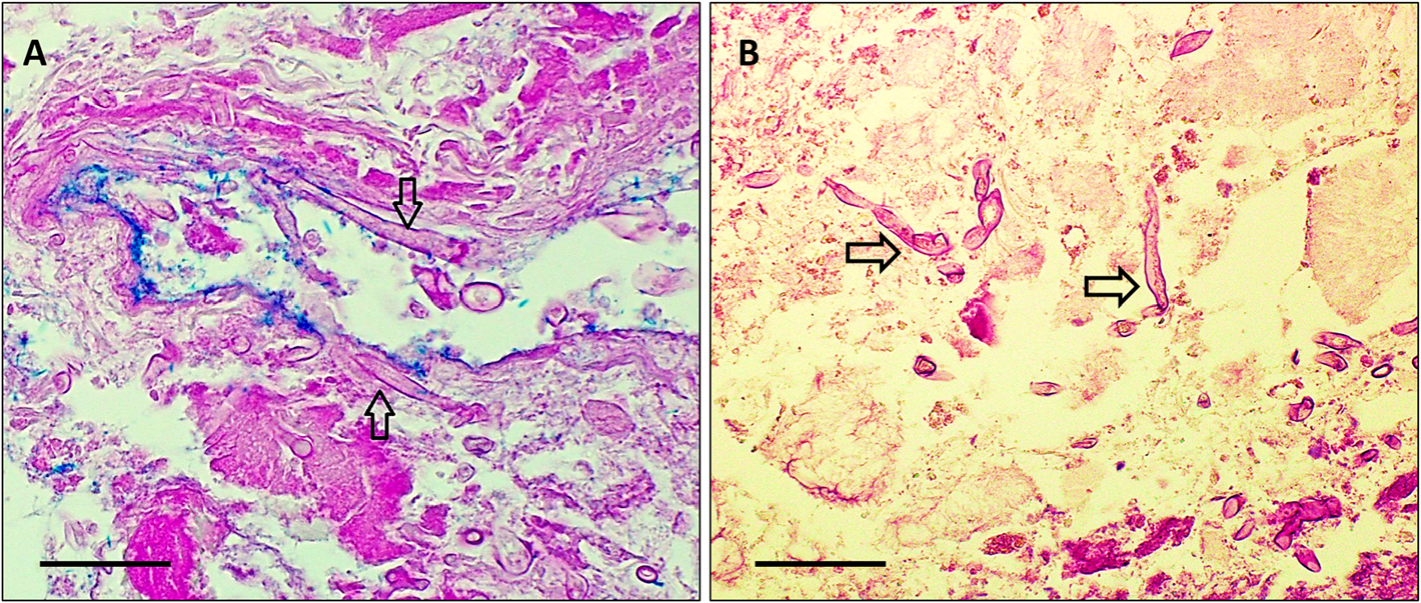
**a** Periodic acid-Schiff stain of skin biopsy from the abdominal wall, demonstrating broad, branching, aseptate fungal hyphae invading a blood vessel wall. **b**. Haematoxylin and eosin stain of a renal biopsy demonstrating broad, aseptate hyphae invading the renal tissue (Bar 20 mm A & B)

Urine microscopy showed > 100 × 10^6^ leucocytes/L, but no pathogen was isolated. Despite antibiotic therapy he had ongoing fevers and persistent flank pain. On day five, he developed an erythematous, indurated lesion on his left upper thigh, and the following day a similar lesion developed on his anterior abdominal wall, with the appearance of ischemic panniculitis (Fig. 1). A repeat CT scan on day 6 demonstrated absent perfusion in the lower pole of the left kidney, consistent with infarction (Fig. 2). A skin lesion biopsy was performed 2 days later, and he was commenced on methylprednisolone as treatment for presumed vasculitis. Twenty-four hours later, culture of the skin biopsy demonstrated growth of a fungus with the appearance of an organism of the order *Mucorales* on Sabouraud dextrose agar at 30° and 37 °C. He was commenced on intravenous (IV) liposomal amphotericin B 8 mg/kg daily for treatment of presumed disseminated mucormycosis, and both skin lesions were extensively debrided. Intraoperatively, panniculitis with necrosis was found to extend to the deep fascia. Histologic examination showed fungal hyphae with angioinvasion and infarction of subcutaneous tissue (Fig. 3a). In light of this, all immunosuppression was ceased. Four days later the patient underwent a left nephrectomy, and histologic examination of the kidney revealed necrosis with aseptate, branching hyphae consistent with organisms of the order *Mucorales* (Fig. 3b).

Fungal microscopy with calcofluor staining following 10% potassium hydroxide (KOH) digestion of renal tissue confirmed the presence of wide ribbon-like aseptate fungal hyphae consistent with zygomycete infection. Tissue was cultured on Sabouraud’s dextrose agar which grew a white cottony fungus at 30, 37 and 40 °C on day 3. The isolate was referred to the National Mycology Reference Centre, where the fungus was identified as *Saksenaea* species (sp.) via sequencing of the internal transcribed spacer (ITS1–5.8S-ITS2) region of the ribosomal deoxyribonucleic acid (DNA) and Nucleotide Basic Local Alignment Search Tool (BLASTn) comparison to publicly accessible sequence databases. This demonstrated 93 and 91% sequence similarity to *S. trapezispora* and *S. oblongispora* Type material, respectively, in both the National Center for Biotechnology Information (NCBI) database (https://blast.ncbi.nlm.nih.gov/) and the Westerdijk Fungal Biodiversity Institute database (http://www.wi.knaw.nl/Collections/BioloMICSSequences.aspx) [6], and 81% similarity to *S. vasiformis* using the International Society of Human and Animal Mycology (ISHAM) barcoding database (http://its.mycologylab.org/). This was considered insufficient for definitive species identification. Further investigation of the species identification, including the possibility of a novel species, will be conducted. The isolate failed to sporulate after 6 weeks of incubation, despite the use of a range of sporulation-inducing media including Potato Dextrose agar and Tap Water agar at 25 °C and 35 °C; therefore antifungal susceptibility testing could not be performed. The patient received 6 weeks of IV amphotericin; oral posaconazole was added 3 weeks into therapy, though significant improvement had been observed following surgical intervention. Posaconazole was given in the form of delayed release tablets 300 mg, with a 12-h interval between the first two doses and daily dosing thereafter. His treatment course was complicated by acute kidney injury likely secondary to IV liposomal amphotericin requiring dose reduction, and abnormal liver function tests likely secondary to posaconazole. He was discharged home after 57 days in hospital on oral posaconazole treatment. Therapeutic drug monitoring of posaconazole was performed and levels were considered therapeutic between 1.5 and 3.3 mg/L.

Three months following discharge, he represented with dyspnoea, rhinorrhoea and cough. CT demonstrated consolidation in the lower lobe of the left lung and upper lobe opacities with inflammatory change in the sinuses. Bronchoscopy was unremarkable and fungal culture was negative. This presentation was thought to be secondary to a flare of eosinophilic granulomatosis with polyangiitis in the setting of cessation of his immunosuppression. He received prednisone, which was slowly weaned, and was later switched to mepolizumab (an interleukin-5 inhibitor) for ongoing treatment of EGPA. The patient has received 18 months of posaconazole treatment with no evidence of recurrence of infection, and we remain cautiously optimistic.

## Discussion and conclusions

We describe a case of disseminated mucormycosis secondary to infection with *Saksenaea* sp*.* with an unusual presentation and good outcome to date. Genus *Saksenaea* was first described in 1953, and due to lack of sporulation during growth in the laboratory has been reported rarely. Failure to sporulate on most occasions means that antifungal susceptibility testing cannot be performed, and treatment is usually based on minimum inhibitory concentration (MIC) data for related species of the order *Mucorales* [7]. There have been reports of higher MICs for Amphotericin B and lower MICs for posaconazole for *Saksenaea* spp. than for *Mucor* spp. [8, 9]. In an experimental murine model of disseminated *Saksenaea vasiformis* infection, posaconazole demonstrated the greatest in vitro activity and was the most effective in prolonging survival in this model [10]. A high dose of 8 mg/kg liposomal amphotericin B was used in this case with early introduction of posaconazole, however there is limited evidence to guide management of *Saksenaea* infections. As for other causes of mucormycosis, timely and (where possible) complete surgical debridement is an integral part of management of *Saksenaea* infections [11].

Despite historical difficulty with laboratory identification, *Saksenaea* spp. are increasingly reported as the cause of human infection, mostly but not limited to tropical and subtropical areas [12]. Unlike other members of the order *Mucorales, Saksenaea* infections usually affect immunocompetent hosts, and are often associated with trauma [13]. To our knowledge, there have been six cases of disseminated *Saksenaea* infection reported; these are summarized in Table 1 [14–19]. Two of these cases had renal involvement, and all six cases had cutaneous lesions. Our case differs in that there has been a favourable outcome to date; survival has been reported in only two cases previously. In those cases, posaconazole was used in combination with IV amphotericin B, whereas treatment in all other cases included IV amphotericin B monotherapy. Due to the unusual presentation in our case, diagnosis of invasive fungal infection was delayed until the skin lesions developed and biopsy was obtained. In three of the previously reported disseminated *Saksenaea* cases, diagnosis was made post-mortem highlighting the challenge of diagnosing this rare entity in a timely fashion. It is important to note, four of these diagnoses were made phenotypically and although they were reported as *Saksenaea vasiformis*, without DNA sequencing, accurate species identification cannot be confirmed.

**Table 1:** Reported cases of disseminated *Saksenaea* spp. infection

Case	Age	Sex	Country/year	Comorbidities	Mode of Infection	Organism	Method of identification	Organ systems involved	Treatment	Outcome
1 [14]	69	Female	United States of America 1981	Acute myeloid leukaemia	Inhalation	*Saksenaea* sp.	Phenotypic	Cutaneous, Pulmonary, Renal	Nil	Died
2 [15]	58	Male	Spain 2014	Type 2 Diabetes Mellitus	Trauma	*Saksenaea vasiformis complex*	Genotypic (ITS sequencing)	Cutaneous, Ocular, Cerebral	Surgical debridement, IV Amphotericin B	Died
3 [16]	59	Male	Australia 2000	Hypertension, Asthma	Inhalation	*Saksenaea* sp.	Phenotypic	Cutaneous, Cardiac, Pulmonary, Thyroid	Surgical debridement, IV Amphotericin B	Died
4 [17]	11	Male	Australia 2008	Nil	Insect bite	*Saksenaea* sp.	Phenotypic	Cutaneous, Renal	Surgical debridement, IV Amphotericin B, Posaconazole	Survived
5 [18]	14	Male	Iraq 1983	Nil	Unknown	*Saksenaea* sp.	Phenotypic	Cutaneous, Pulmonary	Nil	Died
6 [19]	69	Male	Singapore 2020	Hypertension, Ischaemic heart disease	Unknown	*Saksenaea vasiformis complex*	Genotypic (ITS sequencing)	Pulmonary, cutaneous, retroperitoneal lesion	IV Amphotericin B, Posaconazole	Survived

None of the previously reported disseminated *Saksenaea* spp. cases had a history of autoimmune disease or immunosuppressive treatment. In a series of cases of mucormycosis from Australia [20], 9/74 (12%) had a history of an autoimmune or rheumatologic condition such as systemic lupus erythematosus or rheumatoid arthritis, and all but one had received prior treatment with corticosteroids. The infecting fungal species were identified in 7/9, and included *Rhizopus* spp., *Rhizomucor* spp. and *Mucor* spp.; all 9 patients died. Despite a history of EPGA with immunosuppressive treatment, our patient survived. We attribute the good outcome to aggressive surgical debridement and combination antifungal treatment with liposomal amphotericin and posaconazole.

In summary, species of the genus *Saksenaea* are emerging as fungal pathogens with worldwide distribution, causing mucormycosis in both immunocompetent and immunocompromised hosts. Making the diagnosis can be challenging due to diverse clinical manifestations and difficulty with phenotypic identification, and susceptibility information is often elusive. We describe an unusual case of disseminated infection with this organism, associated with a good outcome 18 months after presentation.

## Data Availability

Data sharing is not applicable to this article as no datasets were generated or analysed during the current study.

## Abbreviations

BLASTn: Nucleotide Basic Local Alignment Search Tool
CT: Computed Tomography
DNA: Deoxyribonucleic acid
EGPA: Eosinophilic granulomatosis with polyangiitis
ISHAM: International Society of Human and Animal Mycology
ITS: Internal Transcribed Spacer
IV: Intravenous
KOH: Potassium hydroxide
MIC: Minimum Inhibitory Concentration
NCBI: National Center for Biotechnology Information
spp.: species’
sp.: species

## References

[1] Gomes MZ, Lewis RE, Kontoyiannis DP (2011). Mucormycosis caused by unusual mucormycetes, non-Rhizopus, −Mucor, and -Lichtheimia species. Clin Microbiol Rev.

[2] Saksena SB (1953). A new genus of the Mucorales. Mycologia..

[3] Walther G, Wagner L, Kurzai O (2019). Updates on the Taxonomy of Mucorales with an Emphasis on Clinically Important Taxa. J Fungi (Basel).

[4] Labuda R, Bernreiter A, Hochenauer D, Schuller C, Kubatova A, Strauss J (2019). Saksenaea dorisiae sp. nov., a new opportunistic pathogenic fungus from Europe. Int. J Microbiol.

[5] Alvarez E, Garcia-Hermoso D, Sutton DA, Cano JF, Stchigel AM, Hoinard D (2010). Molecular phylogeny and proposal of two new species of the emerging pathogenic fungus Saksenaea. J Clin Microbiol.

[6] Coordinators NR (2016). Database resources of the National Center for biotechnology information. Nucleic Acids Res.

[7] Ellis DH, Kaminski GW (1985). Laboratory identification of Saksenaea vasiformis: a rare cause of zygomycosis in Australia. Sabouraudia..

[8] Blanchet D, Dannaoui E, Fior A, Huber F, Couppie P, Salhab N (2008). Saksenaea vasiformis infection, French Guiana. Emerg Infect Dis.

[9] Sun QN, Fothergill AW, McCarthy DI, Rinaldi MG, Graybill JR (2002). In vitro activities of posaconazole, itraconazole, voriconazole, amphotericin B, and fluconazole against 37 clinical isolates of zygomycetes. Antimicrob Agents Chemother.

[10] Salas V, Pastor FJ, Calvo E, Sutton D, Garcia-Hermoso D, Mayayo E (2012). Experimental murine model of disseminated infection by Saksenaea vasiformis: successful treatment with posaconazole. Med Mycol.

[11] Spellberg B, Ibrahim AS (2010). Recent advances in the treatment of mucormycosis. Curr Infect Dis Rep.

[12] Prakash H, Chakrabarti A (2019). Global Epidemiology of Mucormycosis. J Fungi (Basel).

[13] Samaras K, Markantonatou AM, Karapiperis D, Digonis P, Kartalis N, Kostogloudis N (2019). Saksenaea vasiformis infections: a case of an immunocompetent adult after mild injury and a literature review. J Mycol Med.

[14] Torell J, Cooper BH, Helgeson NG (1981). Disseminated Saksenaea vasiformis infection. Am J Clin Pathol.

[15] Gómez-Camarasa C, Rojo-Martín MD, Miranda-Casas C, Alastruey-Izquierdo A, Aliaga-Martínez L, Labrador-Molina JM (2014). Disseminated infection due to Saksenaea vasiformis secondary to cutaneous Mucormycosis. Mycopathologia..

[16] Solano TAB, Tambosis E, Mann S, Gottlieb T (2000). Disseminated Mucormycosis due to Saksenaea vasiformis in an Immunocompetent adult. Clin Infect Dis.

[17] Trotter DJ, Gonis G, Cottrill E, Coombs C (2008). Disseminated Saksenaea vasiformis in an immunocompetent host. Med J Aust.

[18] Hay RCC, Marshall W, Rees B, Pincott J (1983). Disseminated zygomycosis (mucormycosis) caused by Saksenaea vasiformis. J Infect.

[19] Liang En W, Seow Yen T, Ai Ling T, Yen Ee T, Sze Hwa T, Chun AC (2020). Disseminated Mucormycosis due to Saksenaea vasiformis complex in an Immunocompetent adult with sustained response to Posaconazole treatment. Mycopathologia..

[20] Kennedy KJ, Daveson K, Slavin MA, van Hal SJ, Sorrell TC, Lee A (2016). Mucormycosis in Australia: contemporary epidemiology and outcomes. Clin Microbiol Infect.

